# Prescription drug monitoring programs use mandates and prescription stimulant and depressant quantities

**DOI:** 10.1186/s12889-023-16256-9

**Published:** 2023-07-11

**Authors:** Christian Gunadi, Yuyan Shi

**Affiliations:** Herbert Wertheim School of Public Health and Human Longevity Science, University of California San Diego, 9500 Gilman Drive, La Jolla, CA 92093-0628 USA

**Keywords:** Health policy/regulation, Substance abuse, Observational data/quasi-experiment

## Abstract

**Background:**

While the mandate to check patients’ prescription history in Prescription Drug Monitoring Program (PDMP) database before prescribing/dispensing controlled drugs has been shown to be an important tool to curb opioid abuse, less is known about whether the mandate can reduce the misuse of other commonly abused prescription drugs. We examined whether PDMP use mandates were associated with changes in prescription stimulant and depressant quantities.

**Methods:**

Using data from Automated Reports and Consolidate Ordering System (ARCOS), we employed difference-in-differences design to estimate the association between PDMP use mandates and prescription stimulant and depressant quantities in 50 U.S. states and the District of Columbia from 2006 to 2020. Limited PDMP use mandate was specific only to opioids or benzodiazepines. Expansive PDMP use mandate was non-specific to opioid or benzodiazepine and required prescribers/dispensers to check PDMP when prescribing/dispensing targeted controlled substances in Schedule II-V. The main outcomes were population-adjusted prescription stimulant (amphetamine, methylphenidate, lisdexamfetamine) and depressant (amobarbital, butalbital, pentobarbital, secobarbital) quantities in grams.

**Results:**

There was no evidence that limited PDMP use mandate was associated with a reduction in the prescription stimulant and depressant quantities. However, expansive PDMP use mandate that was non-specific to opioid or benzodiazepine and required prescribers/dispensers to check PDMP when prescribing/dispensing targeted controlled substances in Schedule II-V was associated with 6.2% (95% CI: -10.06%, -2.08%) decline in prescription amphetamine quantity.

**Conclusion:**

Expansive PDMP use mandate was associated with a decline in prescription amphetamine quantity. Limited PDMP use mandate did not appear to change prescription stimulant and depressant quantities.

**Supplementary Information:**

The online version contains supplementary material available at 10.1186/s12889-023-16256-9.

## Background

Drug overdose is an epidemic in the United States. Close to 841,000 people died from drug overdose from 1999 to 2019 [1]. Individuals misusing prescription drugs or drug misusers are at risk of fatal and non-fatal overdose [2, 3]. Drug misusers may obtain prescription drugs for nonmedical purposes through multiple prescriptions from multiple prescribers (“Doctor shopping”) or from friends or family members for whom the drugs were prescribed (“Drug Diversion”) [4, 5]. In 2020, more than 16 million Americans reported misuse of prescription psychotherapeutics such as pain relievers, stimulants, and sedatives [6].

As a way to combat misuse of prescription drugs, states have turned to Prescription Drug Monitoring Program (PDMP). PDMP is a statewide database that records patients’ prescriptions for controlled drugs, allowing health providers to check patients’ prescription patterns that is consistent with doctor shopping or drug diversion behaviors. As of 2020, all but one state (Missouri) have implemented PDMP. Prior to PDMP use mandates, however, only a small fraction of providers enrolled and checked patients’ prescriptions history in PDMP [7–9]. This low participation rate can be attributed to the voluntary nature of accessing PDMP prior to prescribing or dispensing controlled substances.

To boost the utilization rate of PDMP, many states have recently started mandating the use of PDMP prior to prescribing/dispensing controlled drugs. Evidence from early states mandating PDMP use indicated that the utilization rate of PDMP rose substantially after the mandate. Following PDMP use mandate in 2012, the number of prescription history reports queried by providers in Kentucky increased by over 230% from 811,000 to 2011 to 2,691,000 in 2012 [9]. Similar increase was also observed in Tennessee, New York, and Ohio after these states implemented PDMP use mandate [9].

Unsurprisingly, early research on PDMP without use mandate found limited or no evidence of an effect on prescription drug abuse [10–12]. On the contrary, recent studies examining the PDMP use mandate, which was primarily designed to reduce opioid abuse and therefore the focus of these studies, show that the mandate is a promising tool to reduce opioid prescriptions. Using Medicaid prescription data across the U.S. states from 2011 to 2016, Wen et al. [13] suggested that PDMP use mandate was associated with a reduction in the opioid prescription rate from 161.47 to 147.07 per 1,000 Medicaid enrollees. Similarly, Buchmueller and Carey [14] found an 8% reduction in the percentage of Medicare Part D enrollees who obtain prescriptions from five or more prescribers. Zhang et al. [15] found that PDMP use mandate was associated with an 11–15% reduction in opioids dispensed to patients with sickle cell disease or cancer with bone metastasis. Stein et al. [16] showed that PDMP use mandate was associated with a significant reduction in initial high-risk opioid prescribing (> 7 days). With some exceptions (e.g., Brown et al. [17]), most of recent studies have shown that PDMP use mandate was linked to reduced opioid prescriptions [10, 18–23].

While emerging research on PDMP use mandate has mainly focused on opioids, fewer studies examine whether the mandate has the potential to reduce drug misuse for other controlled substances. Indeed, recent statistics have shown the importance of examining whether state-level regulations, such as PDMP use mandate, can decrease the misuse of stimulants and depressants drugs. For example, Hoots et al. [24] found that psychostimulant-involved death without opioids increased by 22.6% per year from 2008 to 2017. Bachhuber et al. [25] found that Benzodiazepine overdose death rate increased by almost six times between 1996 and 2013. The deaths due to non-benzodiazepine hypnotic/sedatives had also increased significantly between 2000 and 2018 [26].

A few studies examining the relationship between PDMP use mandate and the misuse of prescription drugs other than opioids have so far yielded mixed results [10, 27–29]. Analyzing eight U.S. states that implemented PDMP use mandate between 2000 and 2013, Meinhofer [10] found that PDMP use mandate reduced prescription stimulant quantity by 11%. Similarly, based on 24 U.S. states that implemented PDMP use mandate between 2009 and 2017, Beheshti and Kim [27] found that PDMP use mandate decreased prescription stimulant quantity by 16.6%. Winstanley et al. [29] show that there was a significant reduction in the benzodiazepine quantity dispensed in Ohio after the passage of House Bill 341, which mandated the use of Ohio’s PDMP. On the contrary, a national study by Liang et al. [28] did not find evidence that PDMP use mandate reduced benzodiazepine prescribing among Medicaid enrollees.

In this study, we examined the association between PDMP use mandates and prescription stimulant and depressant quantities, which are two classes of commonly abused non-opioid prescription drugs [30]. We expanded previous studies by considering more recent data (2006–2020 for stimulants and 2006–2017 for depressants due to data availability); while Meinhofer [10] and Beheshti and Kim [27] analysis was based on eight and 24 states that implemented PDMP use mandate as of 2013 and 2017, respectively, our analysis was based on 44 states that implemented the mandate as of 2020. We also considered variations in PDMP use mandates. Some states only require health providers to check PDMP prior to prescribing/dispensing opioids or benzodiazepines, while others are non-specific to opioids or benzodiazepines and require health providers to check PDMP prior to prescribing/dispensing targeted controlled substances in Drug Enforcement Agency Schedule II-V.

## Methods

### Data sources

The prescription drug data were obtained from the Drug Enforcement Administration’s Automation of Reports and Consolidated Orders System (ARCOS) [31]. Following the passage of the Controlled Substances Act of 1970, manufacturers and distributors of controlled substances are required to report their controlled substances transactions to the DEA. ARCOS is a drug reporting system in which manufacturers and distributors report their controlled substance transactions to the Drug Enforcement Administration (DEA). It reports the quantities (in grams) of drugs purchased by hospitals, retail pharmacies, practitioners, mid-level practitioners, and teaching institutions. For the analysis, we relied on ARCOS Report 2, which reported drug quantities quarterly at the state level.

We examined prescription stimulant and depressant quantities that were consistently reported by ARCOS throughout the period of analysis. Stimulant drugs included Amphetamine, Methylphenidate, and Lisdexamfetamine, and depressants included Barbiturates (Amobarbital, Pentobarbital, Butalbital, and Secobarbital). The period of analysis for Amphetamine and Methylphenidate was 2006–2020. 2006 was the starting year because the data on Amphetamine before 2006 was not comparable to after 2006. ARCOS data for the 2021 reporting year was still incomplete at the time of analysis. For Lisdexamfetamine, data was only available from the second quarter of the year 2007, so the period of analysis was from the second quarter of 2007 to the fourth quarter of 2020. Barbiturates were not consistently reported in ARCOS before 2006. Additionally, 2017 is the most recent year available. Therefore, the period of study for depressants was 2006–2017.

### Outcome: prescription drug Grams per 100,000

Amphetamine, Methylphenidate, Amobarbital, Lisdexamfetamine, Pentobarbital, Butalbital, and Secobarbital drug quantities were normalized by state population estimated from annual American Community Survey 2006–2020 [32].

### Policy exposure: prescription drug monitoring program (PDMP) use mandate

The policy exposure was the implementation of PDMP use mandate. With some exceptions, the effective dates of PDMP use mandate were obtained from PDMP Training and Technical Assistance Center (PDMP TTAC; see Supplemental Table S1 for details) [33]. Based on the description of the mandate from PDMP TTAC, we divided the PDMP use mandate into two groups: limited and expansive. Limited PDMP use mandate requires prescribers or dispensers to check Prescription Drug Monitoring Program only when prescribing/dispensing opioids or benzodiazepine, while expansive PDMP use mandate is non-specific to opioid/benzodiazepine and requires prescribers or dispensers to check Prescription Drug Monitoring Program when prescribing/dispensing targeted controlled substances in Drug Enforcement Agency Schedule II-V. As an example, Maine required prescribers to query the PDMP upon the initial prescription of an opioid or benzodiazepine medication and every 90 days for as long as the prescription is active, while Alaska required a practitioner to query the PDMP prior to dispensing, prescribing, or administering a Schedule II or III controlled substance except in certain circumstances such as at the scene of an emergency or in an ambulance. As such, we classified Maine as a limited PDMP use mandate state while Alaska was classified as an expansive PDMP use mandate state. We expect the implementation of expansive PDMP use mandate to have a stronger impact on stimulant and depressant drug quantities than the limited PDMP use mandate.

The policy exposure variables were indicators for the presence of limited and expansive PDMP use mandates in the state. Limited PDMP use mandate indicator was coded to one if limited PDMP use mandate was in effect in the state in the period (quarter-year) and zero otherwise. Expansive PDMP use mandate indicator was coded to one if expansive PDMP use mandate was in effect in the state in the period (quarter-year) and zero otherwise. The number of states with PDMP use mandates over the period of the analysis is illustrated in Supplemental Fig. S1.

### Covariates

We considered the following state-level time-varying socioeconomic covariates that may confound the relationship between PDMP use mandates and prescription drug quantities: the share of adults (18+) in the population, the share of population without a high school diploma, the share of non-white individuals in the population, poverty rate, and unemployment rate. These variables were constructed from annual American Community Survey 2006–2020 [32]. Some states also implemented non-mandatory PDMP in the period of analysis. To take this into account, we created an indicator that took the value of one if non-mandatory PDMP was in effect in the state in the period (quarter-year) and zero otherwise. The effective dates for non-mandatory PDMP were obtained from Kim [34].

### Statistical analysis

We conducted the analyses at the state-quarter-year level. In total, we have 3,060 state-quarter-year observations for amphetamine and methylphenidate analysis and 2,805 state-quarter-year observations for lisdexamfetamine analysis. For depressants analysis, we have 2,448 state-quarter-year observations. This difference in the number of observations for stimulants and depressants was due to data availability, as noted above. To estimate the association between PDMP use mandates and prescription drug quantities, we employed a quasi-experimental difference-in-differences research design. Specifically, we utilized log-linear regressions to model the prescription drug grams per 100,000 as a function of PDMP use mandates, adjusting for the covariates as well as state, quarter, and year fixed effects.^1^ The empirical specifications are as follows:

$${\text{ln}(\text{y}}_\text{sqt})=\mathrm\alpha+{\mathrm\delta}_\text{s}+{\mathrm\delta}_\text{q}+{\mathrm\delta}_\text{t}+\mathrm\gamma\text{E}\text{x}\text{p}\text{a}\text{n}\text{s}\text{i}\text{v}\text{e}{\text{P}\text{D}\text{M}\text{P}}_{\text{s}\text{q}\text{t}}+\mathrm\beta{\text{L}\text{i}\text{m}\text{i}\text{t}\text{e}\text{d}\text{P}\text{D}\text{M}\text{P}}_{\text{s}\text{q}\text{t}}+\pi Controls+\varepsilon_{\text{s}\text{q}\text{t}}$$

where ${\text{y}}_{\text{s}\text{q}\text{t}}$ is the outcome in state $\text{s}$ at quarter $\text{q}$ and year $\text{t}$. α is the constant/intercept. ${{\updelta }}_{\text{s}}$, ${{\updelta }}_{\text{q}}$, and ${{\updelta }}_{\text{t}}$ are state, quarter, and year fixed effects, respectively. State fixed effects accounted for unobserved time-invariant state-level confounding factors. Quarter fixed effects accounted for quarterly seasonal differences in prescription drug quantities. Year fixed effects accounted for yearly national-level shocks that apply to all states equally. $\text{L}\text{i}\text{m}\text{i}\text{t}\text{e}\text{d}\text{P}\text{D}\text{M}\text{P}$ is an indicator variable that takes the value of one if limited PDMP use mandate was in effect in state $\text{s}$ at quarter $\text{q}$ and year $\text{t}$ and zero otherwise. $\text{E}\text{x}\text{p}\text{a}\text{n}\text{s}\text{i}\text{v}\text{e}\text{P}\text{D}\text{M}\text{P}$ is an indicator variable that takes the value of one if expansive PDMP use mandate was in effect in state $\text{s}$ at quarter $\text{q}$ and year $\text{t}$ and zero otherwise. $Controls$ are control variables as described in Covariates subsection above. ${\upepsilon }$ is the error term. We clustered the standard errors at the state level to account for possible serial correlation in the data [35].

Four additional analyses were conducted. First, there might be unobserved regional-level confounding shocks in the period of analysis that influence states’ prescription drug quantities differently across regions. To address this, we added census-division-by-year fixed effects in the model. Second, the main identification assumption of difference-in-differences research design is that there were no unobserved time-varying state-specific factors that are correlated with the timing of PDMP use mandate implementations (i.e., parallel trend assumption). To give support for this assumption, we estimated an event study model, replacing PDMP use mandates indicators with a series of its leads and lags. Finding no discernible differential trends present before PDMP use mandates implementation would bolster the validity of difference-in-differences research design. Third, we conducted a leave-one-out analysis, dropping one PMDP use mandate state from the sample at a time and re-estimating the regression model, to see if our main findings are driven by a specific PDMP use mandate state. Finally, recent studies showed that using two-way fixed effect model to estimate the effect of a policy in which the implementation is staggered is likely to yield a biased estimate in the presence of treatment effect heterogeneity [36–38]. To address this, we conducted a sensitivity analysis by using De Chaisemartin and d’Haultfoeuille estimator that is robust to this concern [36].

## Results

### Descriptive statistics

Supplemental Table S2 reports summary statistics for the baseline period. The year 2006 quarter 1 through 2007 quarter 3 was considered baseline because the first PDMP use mandate implemented in the period of analysis was in quarter 4 of 2007 (Nevada). In the baseline period, there were almost no statistically significant differences in observed characteristics between states implementing limited and expansive PDMP use mandates. An exception was the share of non-white individuals in the population; it was 8% points higher in expansive PDMP use mandate states.

### Difference-in-differences analysis

The regression results from difference-in-differences analysis for stimulants are reported in Table 1. Models 1 and 3 report the estimates without census-division-by-year fixed effects for amphetamine and methylphenidate, respectively. The results indicate that expansive PDMP use mandate was associated with an approximately 5.64% decline (obtained from (${e}^{-0.058}$-1)*100; 95% CI: -9.70%, -1.29%) in amphetamine grams per 100,000. At the same time, there was no evidence that limited PDMP use mandate reduced prescription amphetamine quantity (5.76%; 95% CI: -1%, 12.98%). There was also no evidence that either limited or expansive PDMP use mandate led to a decline in prescription methylphenidate or lisdexamfetamine quantity.

**Table 1:** The Association between Prescription Drug Monitoring Program (PDMP) and prescription stimulant quantity

	ln(Amphetamine Grams per 100,000 Population)		ln(Methylphenidate Grams per 100,000 Population)		ln(Lisdexamfetamine Grams per 100,000 Population)	
	Coefficient (standard error) [95% Confidence Interval]					
	(1)	(2)	(3)	(4)	(5)	(6)
Limited PDMP Use Mandate	0.056	0.027	-0.006	-0.027	0.038	-0.054
	(0.033)	(0.034)	(0.039)	(0.033)	(0.045)	(0.064)
	[-0.010 0.122]	[-0.041 0.094]	[-0.085 0.073]	[-0.093 0.038]	[-0.053 0.129]	[-0.182 0.074]
Expansive PDMP Use Mandate	-0.058*	-0.064**	-0.011	-0.009	-0.034	-0.035
	(0.022)	(0.021)	(0.014)	(0.013)	(0.039)	(0.040)
	[-0.102 -0.013]	[-0.106 -0.021]	[-0.039 0.018]	[-0.035 0.018]	[-0.112 0.045]	[-0.116 0.046]
Include Census-Division-by-Year Fixed Effects?	No	Yes	No	Yes	No	Yes

Notes:

1. Limited PDMP use mandate requires prescribers or dispensers to check PDMP only when prescribing/dispensing opioids or benzodiazepine. Expansive PDMP use mandate is non-specific to opioid/benzodiazepine and requires prescribers or dispensers to check PDMP when prescribing/dispensing targeted controlled substances in Drug Enforcement Agency Schedule II-V

2. All regressions included controls for the share of adults (18+) in the population, the share of the population without a high school diploma, the share of non-white individuals in the population, unemployment rate, poverty rate, non-mandatory PDMP legislation indicator, and state, quarter, and year indicators

3. Standard errors were clustered at the state level

4. The detailed results are reported in Supplemental Table S3

*p < 0.05, **p < 0.01

Table 2 reports the regression results from difference-in-differences analysis for depressants. Focusing on the specification without census-division-by-year fixed effects (Models 1, 3, 5, 7), we found that expansive PDMP use mandate was associated with 8.79% reduction in butalbital prescription grams per 100,000 (95% CI: -15.8%, -1.29%). We did not find evidence that limited PDMP use mandate reduced other depressant quantities.

**Table 2:** The Association between Prescription Drug Monitoring Program (PDMP) and prescription barbiturate quantity

	ln(Amobarbital Grams per 100,000 Population)		ln(Butalbital Grams per 100,000 Population)		ln(Pentobarbital Grams per 100,000 Population)		ln(Secobarbital Grams per 100,000 Population)	
	Coefficient (standard error) [95% Confidence Interval]							
	(1)	(2)	(3)	(4)	(5)	(6)	(7)	(8)
Limited PDMP Use Mandate	0.082	0.051	0.003	0.024	0.119	-0.043	-0.237	-0.473
	(0.142)	(0.149)	(0.129)	(0.089)	(0.141)	(0.078)	(0.271)	(0.300)
	[-0.203 0.366]	[-0.247 0.350]	[-0.256 0.262]	[-0.155 0.203]	[-0.165 0.402]	[-0.200 0.113]	[-0.781 0.307]	[-1.076 0.131]
Expansive PDMP Use Mandate	0.020	0.021	-0.092*	-0.100**	-0.000	-0.016	-0.144	-0.144
	(0.093)	(0.095)	(0.040)	(0.035)	(0.060)	(0.044)	(0.238)	(0.214)
	[-0.167 0.207]	[-0.170 0.211]	[-0.172 -0.013]	[-0.170 -0.030]	[-0.122 0.121]	[-0.105 0.073]	[-0.622 0.333]	[-0.574 0.285]
Include Census-Division-by-Year Fixed Effects?	No	Yes	No	Yes	No	Yes	No	Yes

Notes:

1. Limited PDMP use mandate requires prescribers or dispensers to check PDMP only when prescribing/dispensing opioids or benzodiazepine. Expansive PDMP use mandate is non-specific to opioid/benzodiazepine and requires prescribers or dispensers to check PDMP when prescribing/dispensing targeted controlled substances in Drug Enforcement Agency Schedule II-V.

2. All regressions included controls for the share of adults (18+) in the population, the share of the population without a high school diploma, the share of non-white individuals in the population, unemployment rate, poverty rate, non-mandatory PDMP legislation indicator, and state, quarter, and year indicators

3. Standard errors were clustered at the state level

4. The detailed results are reported in Supplemental Table S4

*p < 0.05, **p < 0.01

### Additional analyses

Models 2 and 4 in Table 1 present the estimates for stimulants when census-division-by-year fixed effects were included in the model specification. Qualitatively similar findings were found. There was evidence that expansive PDMP use mandate was associated with a decline in prescription amphetamine (-6.2% [95% CI: -10.06%, -2.08%]) but not methylphenidate nor lisdexamfetamine quantity. Additionally, there was no evidence that limited PDMP use mandate reduced prescription amphetamine, methylphenidate, or lisdexamfetamine quantities.

Models 1, 3, 5, and 7 in Table 2 report the estimates for depressants when census-division-by-year fixed effects were included in the model specification. Overall, the results were qualitatively similar. We found that expansive PDMP use mandate was associated with a 9.52% decline (95% CI: -15.63%, -2.96%) in prescription butalbital grams per 100,000. There was no evidence that limited PDMP use mandate reduced other depressant quantities.

Detailed results for difference-in-differences analysis are reported in Supplemental Tables S3 and S4. A plausibly noteworthy result was that non-mandatory PDMP legislation was associated with a decline in prescription amphetamine quantity. However, this estimate was no longer statistically significant when census-division-by-year fixed effects were included in the model (-4.69%, 95% CI [-9.88%, 0.9%]).

**Fig. 1:**
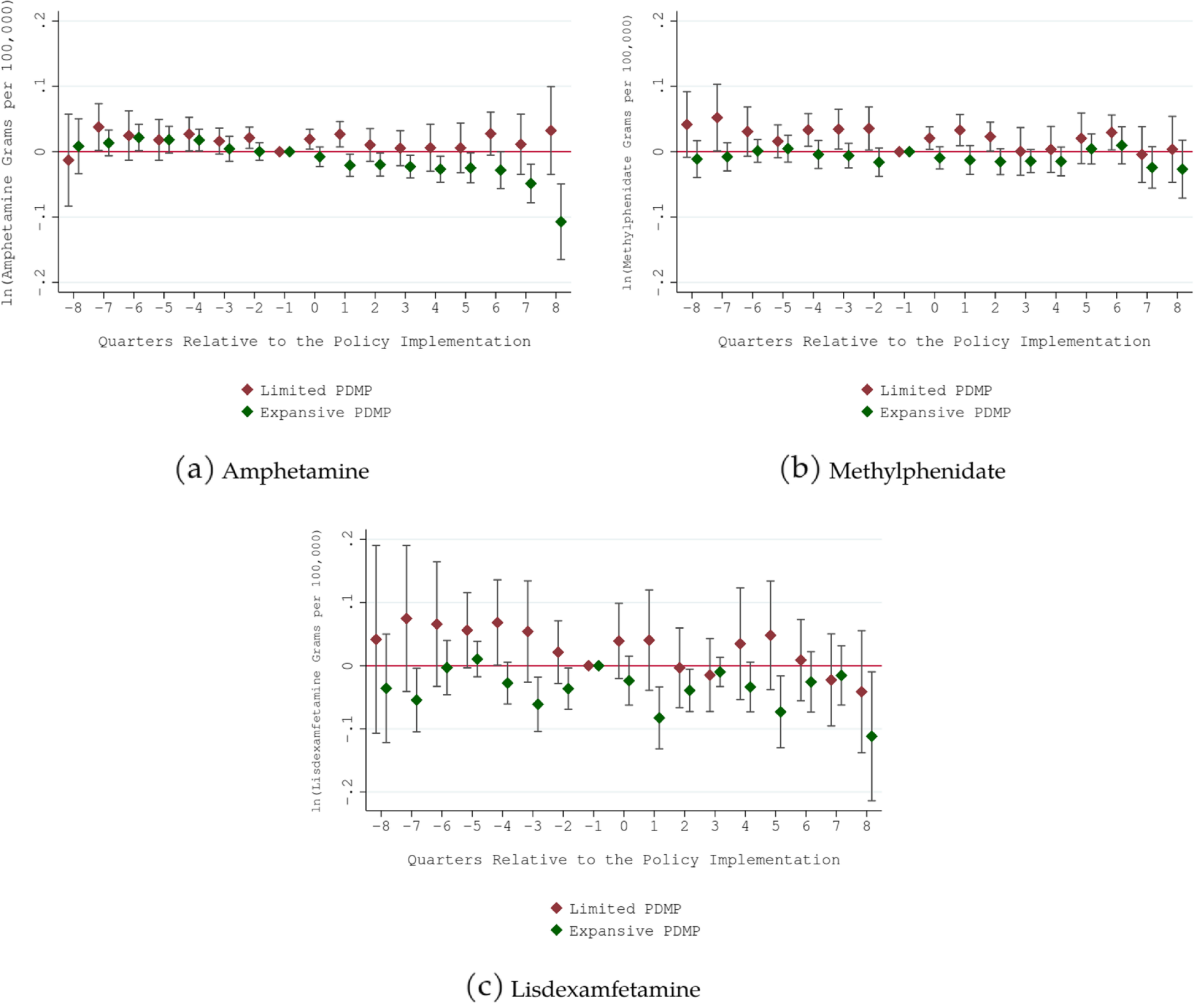
Event Study for Prescription Stimulant Grams per 100,000 Population. Notes: 1. Limited PDMP use mandate requires prescribers or dispensers to check Prescription Drug Monitoring Program only when prescribing/dispensing opioids or benzodiazepine. Expansive PDMP use mandate is non-specific to opioid/benzodiazepine and requires prescribers or dispensers to check Prescription Drug Monitoring Program when prescribing/dispensing targeted controlled substances in Drug Enforcement Agency Schedule II-V. 2. Estimated coefficient and 95% CIs are reported. 3. The quarter prior to the implementation was the reference (omitted) quarter. The estimated coefficient should be interpreted as relative to this quarter. The final lag/lead points accumulated all quarters beyond (i.e., -8 captured quarter − 8 and earlier; 8 captured quarter 8 and later). 4. All regressions included controls for the share of adults (18+) in the population, the share of the population without a high school diploma, the share of non-white individuals in the population, unemployment rate, poverty rate, non-mandatory PDMP legislation indicator, and state, quarter, year, and census-division-by-year indicators. 5. Standard errors were clustered at the state level

**Fig. 2:**
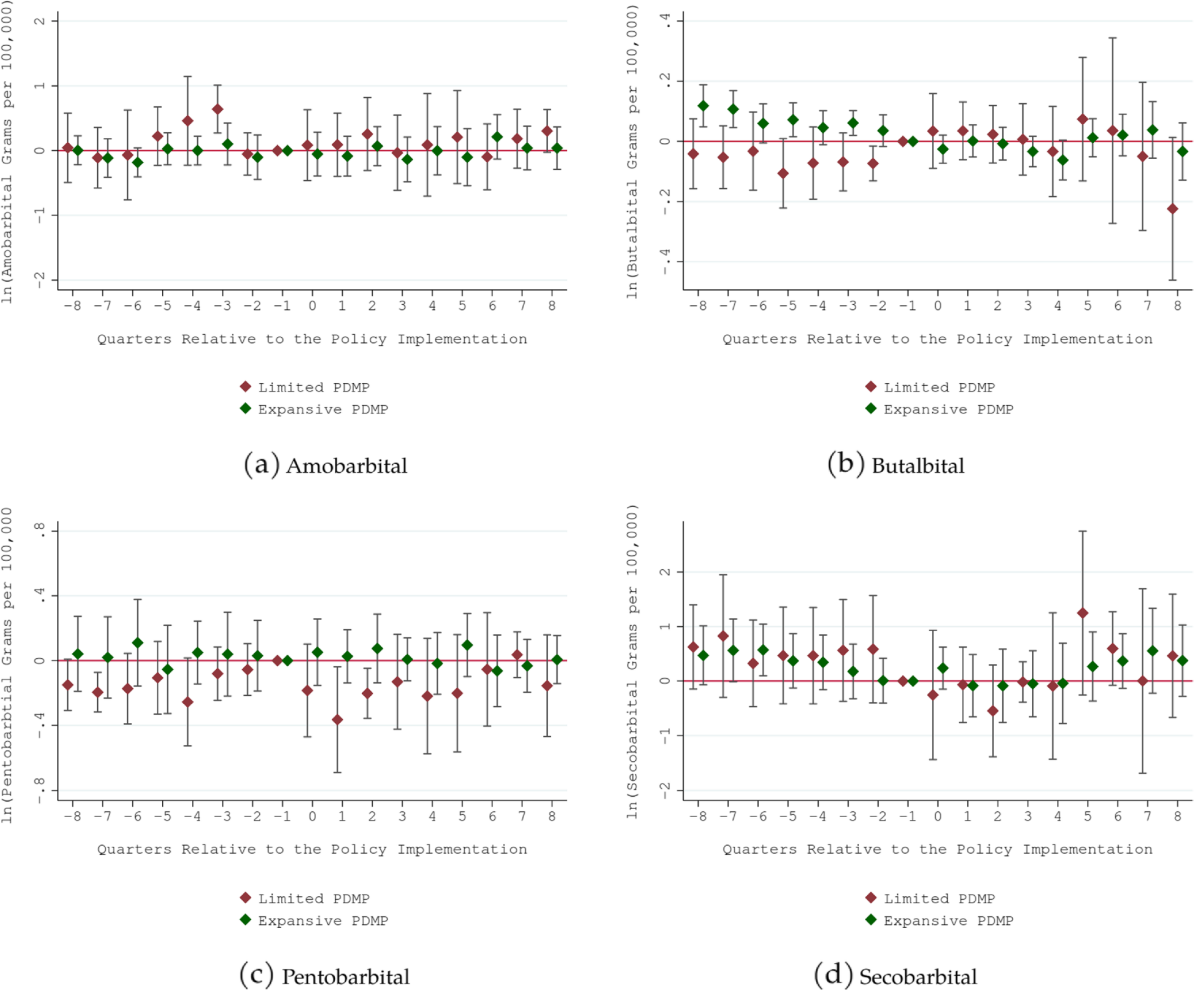
Event Study for Prescription Barbiturate Grams per 100,000 Population. Notes: 1. Limited PDMP use mandate requires prescribers or dispensers to check Prescription Drug Monitoring Program only when prescribing/dispensing opioids or benzodiazepine. Expansive PDMP use mandate is non-specific to opioid/benzodiazepine and requires prescribers or dispensers to check Prescription Drug Monitoring Program when prescribing/dispensing targeted controlled substances in Drug Enforcement Agency Schedule II-V. 2. Estimated coefficient and 95% CIs are reported. 3. The quarter prior to the implementation was the reference (omitted) quarter. The estimated coefficient should be interpreted as relative to this quarter. The final lag/lead points accumulated all quarters beyond (i.e., -8 captured quarter − 8 and earlier; 8 captured quarter 8 and later). 4. All regressions included controls for the share of adults (18+) in the population, the share of the population without a high school diploma, the share of non-white individuals in the population, unemployment rate, poverty rate, non-mandatory PDMP legislation indicator, and state, quarter, year, and census-division-by-year indicators. 5. Standard errors were clustered at the state level

Figures 1 and 2 show the results from event studies. In most cases, we did not find evidence that differential trends prior to the implementation of PDMP use mandates were driving the findings. An exception was prescription butalbital quantity. There was a notable downward trend in prescription butalbital quantity prior to the implementation of expansive PDMP use mandate, indicating that the negative significant association in Table 2 reflect this pre-policy trend rather than the real impact of expansive PDMP use mandate.

Supplemental Figs. S2 and S3 show the results from leave-one-out analysis. Except for a few cases, the estimates were largely consistent with the main results. Overall, there was a lack of evidence that the main findings were systematically driven by the inclusion of a specific state in the sample.

Finally, Supplemental Figs. S4 and S5 show the results based on De Chaisemartin and d’Haultfoeuille estimator. Overall, the results qualitatively hold.

## Discussion

This study shows that expansive PDMP use mandate was associated with a decline (~ 6%) in prescription amphetamine quantity. We did not find evidence that prescription methylphenidate quantity was reduced by expansive PDMP use mandate. These findings are consistent with Meinhofer [10], which found that PDMP use mandate reduced aggregated prescription stimulant quantity (amphetamine and methylphenidate combined) by about 11% based on the experience of eight states that implemented the mandate by 2013. However, since our analysis was not aggregated across stimulants, our findings indicated that the estimated association between PDMP use mandate and stimulants in Meinhofer [10] is likely to be coming from a decline in amphetamine rather than methylphenidate quantity.

Our findings also suggest that expansive PDMP use mandate was associated with about 10% decrease in prescription butalbital quantity. However, the event study result shows that prescription butalbital quantity was already declining even before the policy was implemented. This finding implies that the estimated association between expansive PDMP use mandate and prescription butalbital quantity reflected unobserved time-varying confounding factors rather than causal policy impact. We did not find evidence that expansive PDMP use mandate was associated with changes in other depressant quantities. Additionally, we also did not find evidence that limited PDMP use mandate was associated with a decline in prescription stimulant and depressant quantities. This finding is unsurprising, mainly because limited PDMP use mandate is quite specific, requiring prescribers or dispensers to check PDMP only when they prescribe/dispense opioids or benzodiazepine.

While emerging studies on PDMP use mandate have focused on its potency to reduce opioid misuse, less attention has been given to whether the mandate can curb misuse of other commonly abused prescription drugs. So far, the research on this has yielded mixed findings [10, 28, 29]. We contributed to this literature by providing a new set of results from 44 states that implemented PDMP use mandates as of 2020. Our findings are relatively nuanced. We did not find evidence that limited PDMP use mandate was associated with prescription stimulant and depressant quantities. Additionally, although expansive PDMP use mandate was associated with a decline in prescription amphetamine quantity, there was no evidence that it reduced the quantity of other prescription drugs considered in this study. While the results for limited PDMP use mandate might be as expected, it is unclear why expansive PDMP use mandate appeared to be ineffective in reducing the prescription drug quantities other than amphetamine. Differences in the clinical use of different stimulant drugs might play a role in this result. We hope that future research can shed light on the reasons for this finding.

This study is not without limitations. First, the difference-in-differences design used in the analysis control for time-invariant confounding factors, but unobserved time-varying factors may still confound the estimates. Second, many states implemented PDMP use mandates late in the study period. If the impact of PDMP use mandate takes some time to materialize, the estimated associations may not capture this delayed effect. We encourage future research to replicate our analysis with longer post-period data. Third, ARCOS data did not take into account possible illegal movement of prescription drugs across state lines. More generally, if individuals can obtain prescription drugs through illegal means, the reduction observed in ARCOS data might not reflect an actual decline in prescription drug misuse. This limitation, however, is virtually shared by all studies that used ARCOS data to analyze the association between PDMP use mandate and prescription drug misuse (e.g., Meinhofer [10]). Fourth, ARCOS recorded manufacturers and distributors transactions data and might not reflect the actual use of prescription drugs by users. Fifth, the impact of PDMP use mandates may vary across sociodemographic groups. The aggregated nature of ARCOS data did not allow us to conduct the analysis separately along these lines. Six, since PDMP is mainly designed to reduce opioid abuse, patients’ prescription history with regard to opioids might be recorded more comprehensively compared to other prescription drugs in PDMP. Our analysis did not take into account the possibility that patients’ prescription history regarding stimulants and depressants might be recorded less comprehensively than opioids in states that implemented PDMP use mandates. Finally, the analysis did not consider the strength of PDMP mandates (i.e., awareness about the legislation or how strict the mandates were enforced). If prescribers/dispensers did not check PDMP consistently due to lax enforcement/awareness, the mandates would have a limited impact on stimulant/depressant quantities. PDMP use mandate also has other defining features, such as applicable types of prescribers (e.g., all specialties or pain medicine only), the setting in which they practice (e.g., all settings or pain clinics only), and the extent of prescriber discretion allowed, that might play important roles in determining the effectiveness of PDMP mandates. We encourage future research to examine other features of PDMP mandates not covered in the current analysis.

## Conclusion

Recent works have shown that PDMP use mandate is an important tool to curb opioid abuse [13–16]. Less is known, however, on the ability of PDMP use mandate in reducing the misuse of other commonly abused prescription drugs. This study is one of the first few studies that examine the association between PDMP use mandates and prescription drug quantities other than opioids. The results of the analysis indicated that expansive PDMP use mandate, which was non-specific to opioid or benzodiazepine and required prescribers/dispensers to check PDMP when prescribing/dispensing targeted controlled substances in DEA Schedule II-V, was associated with an approximately 6.2% decline in prescription amphetamine quantity. Implementing PDMP use mandate that was non-specific to opioid or benzodiazepine may help efforts to curb amphetamine abuse.

## Supplementary Information

**Additional file 1: Table S1.** Prescription Drug Monitoring Program (PDMP) Legislations. **Figure S1.** Number of States with Prescription Drug Monitoring Programs (PDMP) Use Mandate Over Time. **Table S3.** The Association between Prescription Drug Monitoring Program (PDMP) and Prescription Stimulant Quantity. **Table S4.** The Association between Prescription Drug Monitoring Program (PDMP) and Prescription Barbiturate Quantity. **Figure S2.** Leave-one-out Analysis (Stimulants). **Figure S3.** Leave-one-out Analysis (Barbiturates). **Figure S4.** Sensitivity Analysis Using De Chaisemartin and d’Haultfoeuille (2020) Estimator (Stimulants). **Figure S5.** Sensitivity Analysis Using De Chaisemartin and d’Haultfoeuille (2020) Estimator (Depressants).

**Additional file 2.**

## Data Availability

The dataset supporting the conclusions of this article is included within the article.

## Abbreviations

ARCOS: Automated Reports and Consolidated Ordering System
DEA: Drug Enforcement Agency
PDMP: Prescription Drug Monitoring Program
PDMP TTAC: Prescription Drug Monitoring Program Training and Technical Assistance Center

## Footnotes

1 To avoid dropping observations with zero values, we added 1 to the prescription drug grams before log transformation.
