# Supplementary material for: Prescription drug monitoring programs use mandates and prescription stimulant and depressant quantities
Source: BMC Public Health. 2023 Jul 11;23:1326. doi: 10.1186/s12889-023-16256-9 (PMC10334646; doi:10.1186/s12889-023-16256-9)
Supplement: Supplementary file 1 — Additional file 1: Table S1. Prescription Drug Monitoring Program (PDMP) Legislations. Figure S1. Number of States with Prescription Drug Monitoring Programs (PDMP) Use Mandate Over Time. Table S3. The Association between Prescription Drug Monitoring Program (PDMP) and Prescription Stimulant Quantity. Table S4. The Association between Prescription Drug Monitoring Program (PDMP) and Prescription Barbiturate Quantity. Figure S2. Leave-one-out Analysis (Stimulants). Figure S3. Leave-one-out Analysis (Barbiturates). Figure S4. Sensitivity Analysis Using De Chaisemartin and d’Haultfoeuille (2020) Estimator (Stimulants). Figure S5. Sensitivity Analysis Using De Chaisemartin and d’Haultfoeuille (2020) Estimator (Depressants). [file 12889_2023_16256_MOESM1_ESM.docx]

**SUPPLEMENTAL TABLES AND FIGURES**

**Table S1: Prescription Drug Monitoring Program (PDMP) Legislations**

|  | Non-Mandatory PDMP | Limited PDMP Use Mandate | Expansive PDMP Use Mandate |
| --- | --- | --- | --- |
| Alabama | 2005m11 | 2017m3 |  |
| Alaska | 2008m9 |  | 2017m7 |
| Arizona | 2007m9 |  | 2011m7 |
| Arkansas | 2013m3 | 2015m7 |  |
| California | Prior to 1990 |  | 2018m10 |
| Colorado | 2005m6 | 2018m5 |  |
| Connecticut | 2006m10 |  | 2015m10 |
| Delaware | 2011m9 |  | 2012m3 |
| District of Columbia | 2014m2 | 2021m3 |  |
| Florida | 2010m12 |  | 2018m7 |
| Georgia | 2011m7 |  | 2014m7 |
| Hawaii | Prior to 1990 |  | 2018m7 |
| Idaho | Prior to 1990 | 2020m7 |  |
| Illinois | Prior to 1990 |  | 2018m1 |
| Indiana | Prior to 1990 |  | 2014m7 |
| Iowa | 2006m5 |  | 2018m5 |
| Kansas | 2008m7 |  |  |
| Kentucky | 1998m7 |  | 2012m7 |
| Louisiana | 2006m7 |  | 2014m8 |
| Maine | 2004m1 | 2017m1 |  |
| Maryland | 2011m10 |  | 2018m7 |
| Massachusetts | 1992m12 |  | 2013m1 |
| Michigan | Prior to 1990 |  | 2018m6 |
| Minnesota | 2009m1 |  | 2013m8 |
| Mississippi | 2006m6 |  | 2018m7 |
| Missouri |  |  |  |
| Montana | 2011m7 | 2021m7 |  |
| Nebraska | 2011m8 |  |  |
| Nevada | 1996m1 |  | 2007m10 |
| New Hampshire | 2012m6 | 2017m1 |  |
| New Jersey | 2009m8 | 2015m11 |  |
| New Mexico | 2004m7 |  | 2012m8 |
| New York | Prior to 1990 |  | 2013m8 |
| North Carolina | 2006m1 |  | 2017m6 |
| North Dakota | 2006m12 |  | 2014m10 |
| Ohio | 2005m5 |  | 2011m8 |
| Oklahoma | 1991m1 |  | 2015m11 |
| Oregon | 2009m7 |  | 2021m10 |
| Pennsylvania | Prior to 1990 |  | 2015m6 |
| Rhode Island | Prior to 1990 | 2013m1 |  |
| South Carolina | 2006m6 |  | 2017m5 |
| South Dakota | 2010m3 |  |  |
| Tennessee | 2003m1 |  | 2013m4 |
| Texas | Prior to 1990 |  | 2016m12 |
| Utah | 1995m7 | 2016m5 |  |
| Vermont | 2008m6 |  | 2013m11 |
| Virginia | 2003m9 |  | 2015m7 |
| Washington | 2011m8 |  | 2013m7 |
| West Virginia | 1995m6 |  | 2012m6 |
| Wisconsin | 2010m6 |  | 2017m4 |
| Wyoming | 2003m7 |  | 2019m7 |

Notes:

1. The dates for PDMP use mandates were acquired from the Prescription Drug Monitoring Program Training and Technical Assistance (PDMP TTAC). The effective dates for non-mandatory PDMP were acquired from Kim (2021). The effective date for Delaware PDMP use mandate was not available from PDMP TTAC; the date from Kim (2021) was used. The effective date for Hawaii PDMP use mandate was not available from PDMP TTAC; the date when the law related to the mandate (Hawaii SB 2646) became effective was used (July 1, 2018). The exact date of PDMP use mandate in Mississippi was not reported by PDMP TTAC. The regulation source (<https://pmp.mbp.ms.gov/wp-content/uploads/2019/09/ARTICLE-XLIII-PRESCRIPTION-MONITORING-PROGRAM.pdf>) cited Mississippi Code Annotated Section 73-21-127 that became effective in July 1, 2018 (https://law.justia.com/codes/mississippi/2010/title-73/21/73-21-127/), which we used for the analysis. The effective date for PDMP use mandate in Nevada from PDMP TTAC (October 1, 2017) was not accurate; the effective date of the statute (October 1, 2007) was used for the analysis (<https://www.leg.state.nv.us/Division/Research/Publications/SoL/2007SoL.pdf>). The exact date for PDMP use mandate in Virginia was not reported by PDMP TTAC; the effective date of the statute (July 1, 2015) was used for the analysis (<https://lis.virginia.gov/cgi-bin/legp604.exe?141+ful+CHAP0093>). The effective date for PDMP use mandate in Wyoming was not reported by PDMP TTAC; the effective date of the statute (July 1, 2019) was used for the analysis (<https://legiscan.com/WY/text/SF0047/2019>).
2. Limited PDMP use mandate requires prescribers or dispensers to check Prescription Drug Monitoring Program only when prescribing/dispensing opioids or benzodiazepine. Expansive PDMP use mandate is non-specific to opioid/benzodiazepine and requires prescribers or dispensers to check Prescription Drug Monitoring Program when prescribing/dispensing targeted controlled substances in Drug Enforcement Agency Schedule II-V.

**Figure S1: Number of States with Prescription Drug Monitoring Programs (PDMP) Use Mandate Over Time**

Note: Limited PDMP use mandate requires prescribers or dispensers to check Prescription Drug Monitoring Program only when prescribing/dispensing opioids or benzodiazepine. Expansive PDMP use mandate is non-specific to opioid/benzodiazepine and requires prescribers or dispensers to check Prescription Drug Monitoring Program when prescribing/dispensing targeted controlled substances in Drug Enforcement Agency Schedule II-V.

**Table S2: Summary Statistics for Pooled Quarterly Data in Baseline Period**

|  | Mean [95% Confidence Interval] | | | | |
| --- | --- | --- | --- | --- | --- |
|  | All States | Limited PDMP Use Mandate States | Expansive PDMP Use Mandate States | Differences (Expansive) - (Limited) | |
| Amphetamine Grams per 100,000 Population | 745.15 | 808.64 | 731.29 | -77.35 | [-278.14 123.44] |
| Methylphenidate Grams per 100,000 Population | 1527.22 | 1598.24 | 1499.60 | -98.64 | [-429.39 232.11] |
| Lisdexamfetamine Grams per 100,000 Population | 57.12 | 55.94 | 58.40 | 2.46 | [-12.28 17.21] |
| Amobarbital Grams per 100,000 Population | 0.22 | 0.20 | 0.21 | 0.01 | [-0.08 0.09] |
| Butalbital Grams per 100,000 Population | 246.02 | 233.53 | 245.40 | 11.87 | [-29.01 52.75] |
| Secobarbital Grams per 100,000 Population | 2.02 | 1.98 | 2.05 | 0.07 | [-0.62 0.75] |
| Pentobarbital Grams per 100,000 Population | 891.84 | 836.39 | 885.85 | 49.46 | [-244.77 343.69] |
| Share of Non-White Individuals in the Population | 0.22 | 0.16 | 0.24 | 0.08* | [0.01 0.16] |
| Share of Adults (18+) in the Population | 0.76 | 0.75 | 0.76 | 0.00 | [-0.02 0.02] |
| Share of Population Without High School Diploma | 0.35 | 0.35 | 0.35 | 0.00 | [-0.02 0.03] |
| Poverty Rate | 0.13 | 0.12 | 0.13 | 0.01 | [-0.01 0.03] |
| Unemployment Rate | 0.05 | 0.04 | 0.05 | 0.01 | [-0.00 0.01] |

*p<0.05.

Notes:

1. Limited PDMP use mandate requires prescribers or dispensers to check Prescription Drug Monitoring Program only when prescribing/dispensing opioids or benzodiazepine. Expansive PDMP use mandate is non-specific to opioid/benzodiazepine and requires prescribers or dispensers to check Prescription Drug Monitoring Program when prescribing/dispensing targeted controlled substances in Drug Enforcement Agency Schedule II-V.
2. The summary statistics were based on the baseline period (2006 quarter 1 through 2007 quarter 3). This is the baseline period since the first PDMP mandate occurred in our period of analysis is in quarter 4 of 2007 (Nevada).
3. The differences between limited PDMP use mandate and expansive PDMP use mandate states were obtained by regressing the state characteristics on the indicator for PDMP, with standard errors clustered at the state level to take into account serial correlation in the data.

**Table S3: The Association between Prescription Drug Monitoring Program (PDMP) and Prescription Stimulant Quantity**

|  | **ln(Amphetamine Grams per 100,000 Population)** | | **ln(Methylphenidate Grams per 100,000 Population)** | | **ln(Lisdexamfetamine Grams per 100,000 Population)** | |
| --- | --- | --- | --- | --- | --- | --- |
|  | Coefficient (standard error) [95% Confidence Interval] | | | | | |
|  | (1) | (2) | (3) | (4) | (5) | (6) |
| Limited PDMP Use Mandate | 0.056 | 0.027 | -0.006 | -0.027 | 0.038 | -0.054 |
|  | (0.033) | (0.034) | (0.039) | (0.033) | (0.045) | (0.064) |
|  | [-0.010 0.122] | [-0.041 0.094] | [-0.085 0.073] | [-0.093 0.038] | [-0.053 0.129] | [-0.182 0.074] |
| Expansive PDMP Use Mandate | -0.058* | -0.064** | -0.011 | -0.009 | -0.034 | -0.035 |
|  | (0.022) | (0.021) | (0.014) | (0.013) | (0.039) | (0.040) |
|  | [-0.102 -0.013] | [-0.106 -0.021] | [-0.039 0.018] | [-0.035 0.018] | [-0.112 0.045] | [-0.116 0.046] |
| Non-Mandatory PDMP Legislation | -0.054* | -0.048 | -0.029 | -0.040 | 0.089 | 0.102 |
|  | (0.026) | (0.028) | (0.026) | (0.022) | (0.072) | (0.082) |
|  | [-0.106 -0.002] | [-0.104 0.009] | [-0.081 0.022] | [-0.084 0.003] | [-0.055 0.233] | [-0.061 0.266] |
| Share of Non-White Individuals in the Population | 0.141 | -0.081 | -0.220 | -0.381* | -0.328 | -0.683 |
|  | (0.267) | (0.291) | (0.206) | (0.185) | (0.503) | (0.501) |
|  | [-0.395 0.677] | [-0.666 0.504] | [-0.633 0.194] | [-0.752 -0.010] | [-1.338 0.682] | [-1.689 0.323] |
| Share of Adults (18+) in the Population | 4.372 | 4.021 | 1.118 | 2.131 | 0.411 | -10.507 |
|  | (2.664) | (3.079) | (1.995) | (1.740) | (4.737) | (5.794) |
|  | [-0.978 9.722] | [-2.164 10.206] | [-2.890 5.126] | [-1.364 5.627] | [-9.103 9.925] | [-22.145 1.131] |
| Share of Population Without High School Diploma | 2.556 | 2.645 | 2.255 | 2.381 | 5.198 | 0.650 |
|  | (1.767) | (1.951) | (1.174) | (1.274) | (3.427) | (3.460) |
|  | [-0.993 6.106] | [-1.274 6.563] | [-0.103 4.612] | [-0.177 4.940] | [-1.686 12.082] | [-6.299 7.600] |
| Poverty Rate | -0.730 | -0.860 | -1.311* | -1.455* | -0.616 | -0.889 |
|  | (0.671) | (0.750) | (0.539) | (0.582) | (1.482) | (1.529) |
|  | [-2.079 0.619] | [-2.366 0.646] | [-2.394 -0.227] | [-2.624 -0.286] | [-3.593 2.360] | [-3.960 2.181] |
| Unemployment Rate | -0.912 | -1.482 | -0.111 | 0.147 | 1.157 | 0.003 |
|  | (0.897) | (0.997) | (0.613) | (0.544) | (1.355) | (1.357) |
|  | [-2.713 0.889] | [-3.484 0.520] | [-1.342 1.120] | [-0.946 1.240] | [-1.565 3.880] | [-2.723 2.730] |
| Include Census-Division-by-Year Fixed Effects? | No | Yes | No | Yes | No | Yes |
| R-squared | 0.970 | 0.972 | 0.953 | 0.961 | 0.933 | 0.939 |
| N | 3060 | 3060 | 3060 | 3060 | 2805 | 2805 |

*p<0.05, **p<0.01

Notes:

1. Limited PDMP use mandate requires prescribers or dispensers to check PDMP only when prescribing/dispensing opioids or benzodiazepine. Expansive PDMP use mandate is non-specific to opioid/benzodiazepine and requires prescribers or dispensers to check PDMP when prescribing/dispensing targeted controlled substances in DEA Schedule II-V.
2. All regressions included controls for the share of adults (18+) in the population, the share of the population without a high school diploma, the share of non-white individuals in the population, unemployment rate, poverty rate, non-mandatory PDMP legislation indicator, and state, quarter, and year indicators.
3. Standard errors were clustered at the state level.

**Table S4: The Association between Prescription Drug Monitoring Program (PDMP) and Prescription Barbiturate Quantity**

|  | **ln(Amobarbital Grams per 100,000 Population)** | | **ln(Butalbital Grams per 100,000 Population)** | | **ln(Pentobarbital Grams per 100,000 Population)** | | **ln(Secobarbital Grams per 100,000 Population)** | |
| --- | --- | --- | --- | --- | --- | --- | --- | --- |
|  | Coefficient (standard error) [95% Confidence Interval] | | | | | | | |
|  | (1) | (2) | (3) | (4) | (5) | (6) | (7) | (8) |
| Limited PDMP Use Mandate | 0.082 | 0.051 | 0.003 | 0.024 | 0.119 | -0.043 | -0.237 | -0.473 |
|  | (0.142) | (0.149) | (0.129) | (0.089) | (0.141) | (0.078) | (0.271) | (0.300) |
|  | [-0.203 0.366] | [-0.247 0.350] | [-0.256 0.262] | [-0.155 0.203] | [-0.165 0.402] | [-0.200 0.113] | [-0.781 0.307] | [-1.076 0.131] |
| Expansive PDMP Use Mandate | 0.020 | 0.021 | -0.092* | -0.100** | -0.000 | -0.016 | -0.144 | -0.144 |
|  | (0.093) | (0.095) | (0.040) | (0.035) | (0.060) | (0.044) | (0.238) | (0.214) |
|  | [-0.167 0.207] | [-0.170 0.211] | [-0.172 -0.013] | [-0.170 -0.030] | [-0.122 0.121] | [-0.105 0.073] | [-0.622 0.333] | [-0.574 0.285] |
| Non-Mandatory PDMP Legislation | 0.123 | 0.142 | -0.020 | -0.010 | -0.033 | 0.047 | 0.184 | -0.016 |
|  | (0.113) | (0.111) | (0.042) | (0.034) | (0.075) | (0.057) | (0.212) | (0.183) |
|  | [-0.104 0.351] | [-0.082 0.366] | [-0.105 0.065] | [-0.078 0.059] | [-0.182 0.117] | [-0.068 0.161] | [-0.243 0.610] | [-0.383 0.351] |
| Share of Non-White Individuals in the Population | -1.248 | -1.567 | 1.169 | 1.060 | 2.663 | 2.610 | 1.872 | -0.967 |
|  | (2.652) | (3.239) | (0.695) | (0.882) | (1.695) | (1.722) | (5.834) | (4.974) |
|  | [-6.575 4.078] | [-8.072 4.938] | [-0.228 2.565] | [-0.711 2.832] | [-0.742 6.067] | [-0.850 6.069] | [-9.847 13.590] | [-10.956 9.023] |
| Share of Adults (18+) in the Population | 14.158 | 6.775 | -2.890 | 3.305 | 0.409 | -11.465* | 76.392** | 46.130* |
|  | (9.673) | (9.771) | (4.393) | (4.192) | (6.264) | (5.242) | (25.157) | (22.775) |
|  | [-5.271 33.587] | [-12.851 26.400] | [-11.714 5.935] | [-5.116 11.725] | [-12.173 12.990] | [-21.994 -0.935] | [25.863 126.921] | [0.385 91.875] |
| Share of Population Without High School Diploma | 13.595 | 8.479 | -0.153 | 1.865 | 10.078** | 2.029 | 35.692* | 11.089 |
|  | (7.709) | (6.865) | (2.655) | (2.512) | (3.345) | (2.801) | (16.684) | (17.044) |
|  | [-1.889 29.079] | [-5.309 22.267] | [-5.486 5.179] | [-3.180 6.910] | [3.361 16.796] | [-3.596 7.655] | [2.181 69.202] | [-23.144 45.322] |
| Poverty Rate | -0.053 | 0.150 | -1.059 | -0.595 | -1.744 | -0.636 | -3.806 | -4.814 |
|  | (3.894) | (3.048) | (1.149) | (1.301) | (1.780) | (1.515) | (6.882) | (5.912) |
|  | [-7.875 7.770] | [-5.973 6.273] | [-3.367 1.249] | [-3.208 2.017] | [-5.318 1.830] | [-3.679 2.408] | [-17.628 10.016] | [-16.688 7.061] |
| Unemployment Rate | 3.863 | 2.449 | -0.187 | -1.473 | 1.399 | 2.269 | 13.930 | 18.163** |
|  | (2.434) | (2.993) | (1.469) | (1.561) | (1.533) | (1.466) | (7.959) | (6.327) |
|  | [-1.025 8.751] | [-3.562 8.460] | [-3.137 2.764] | [-4.609 1.662] | [-1.681 4.479] | [-0.675 5.212] | [-2.056 29.917] | [5.455 30.872] |
| Include Census-Division-by-Year Fixed Effects? | No | Yes | No | Yes | No | Yes | No | Yes |
| R-Squared | 0.386 | 0.418 | 0.901 | 0.921 | 0.699 | 0.724 | 0.523 | 0.578 |
| N | 2448 | 2448 | 2448 | 2448 | 2448 | 2448 | 2448 | 2448 |

*p<0.05, **p<0.01

Notes:

1. Limited PDMP use mandate requires prescribers or dispensers to check Prescription Drug Monitoring Program only when prescribing/dispensing opioids or benzodiazepine. Expansive PDMP use mandate is non-specific to opioid/benzodiazepine and requires prescribers or dispensers to check Prescription Drug Monitoring Program when prescribing/dispensing targeted controlled substances in Drug Enforcement Agency Schedule II-V.
2. All regressions included controls for the share of adults (18+) in the population, the share of the population without a high school diploma, the share of non-white individuals in the population, unemployment rate, poverty rate, non-mandatory PDMP legislation indicator, and state, quarter, and year indicators.
3. Standard errors were clustered at the state level.

**Figure S2: Leave-one-out Analysis (Stimulants)**


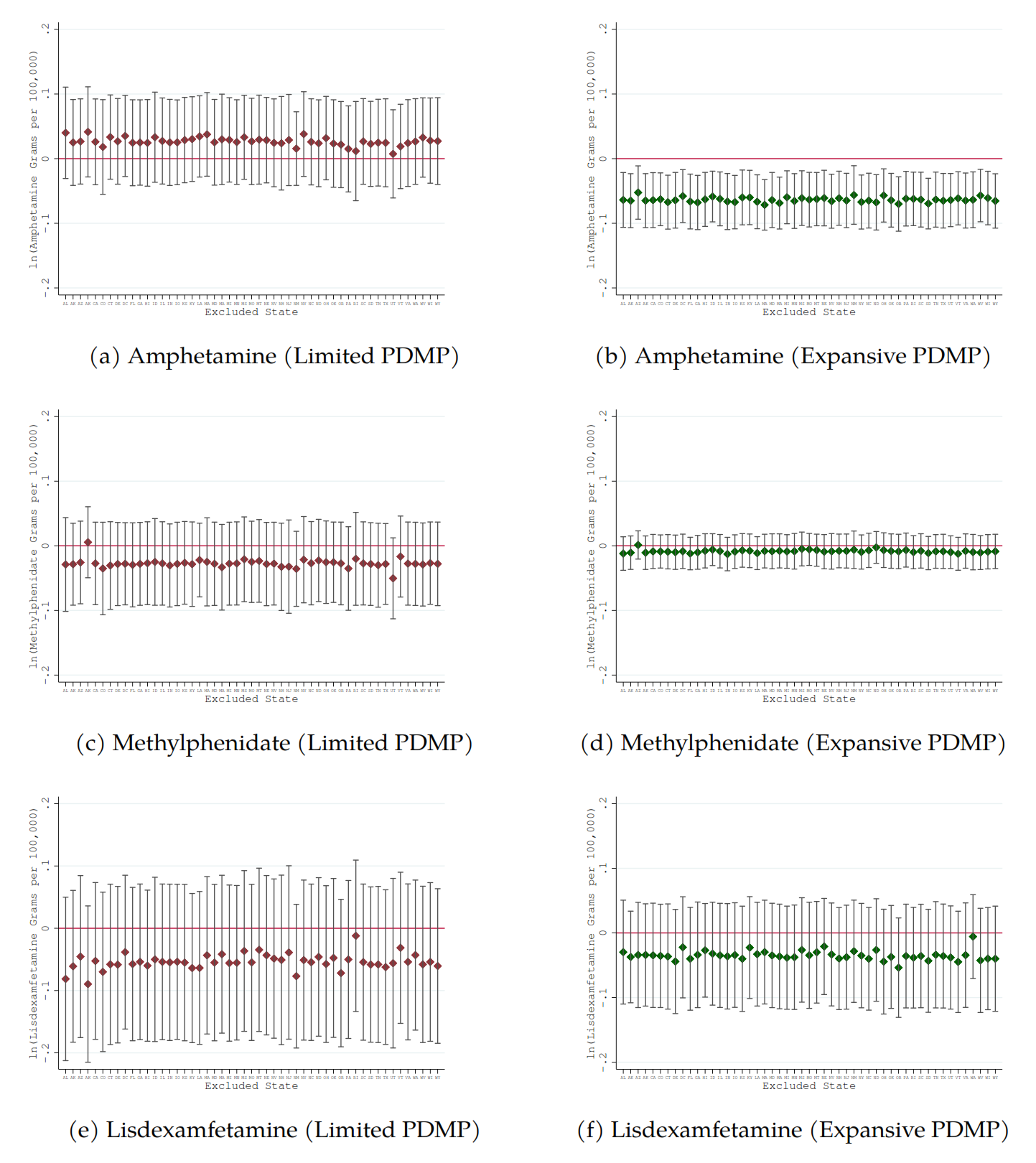


Notes:

1. Limited PDMP use mandate requires prescribers or dispensers to check Prescription Drug Monitoring Program only when prescribing/dispensing opioids or benzodiazepine. Expansive PDMP use mandate is non-specific to opioid/benzodiazepine and requires prescribers or dispensers to check Prescription Drug Monitoring Program when prescribing/dispensing targeted controlled substances in Drug Enforcement Agency Schedule II-V.
2. All regressions included controls for the share of adults (18+) in the population, the share of the population without a high school diploma, the share of non-white individuals in the population, unemployment rate, poverty rate, non-mandatory PDMP legislation indicator, and state, quarter, year, and census-division-by-year indicators.
3. Standard errors were clustered at the state level.

**Figure S3: Leave-one-out Analysis (Barbiturates)**


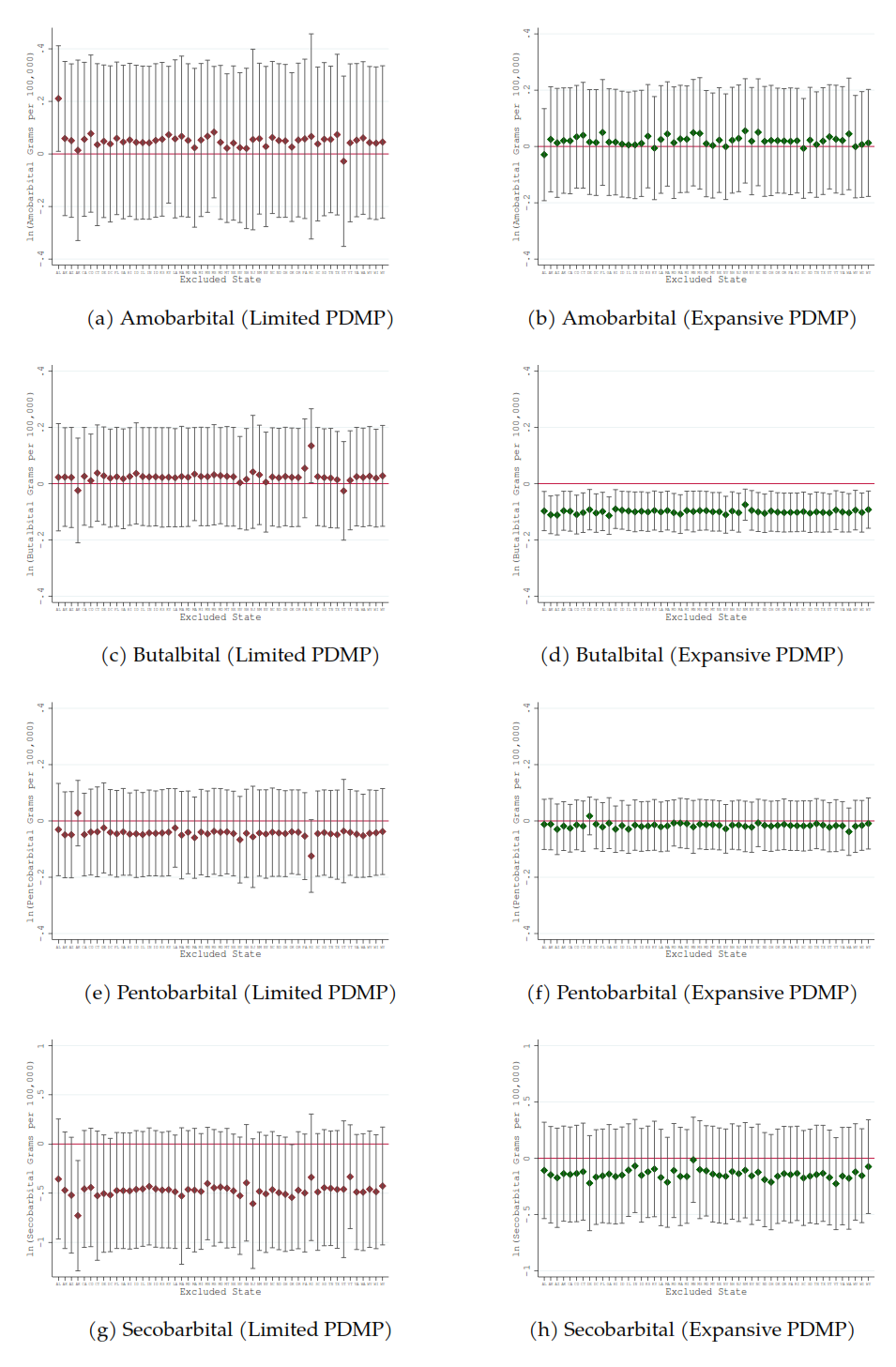


Notes:

1. Limited PDMP use mandate requires prescribers or dispensers to check Prescription Drug Monitoring Program only when prescribing/dispensing opioids or benzodiazepine. Expansive PDMP use mandate is non-specific to opioid/benzodiazepine and requires prescribers or dispensers to check Prescription Drug Monitoring Program when prescribing/dispensing targeted controlled substances in Drug Enforcement Agency Schedule II-V.
2. All regressions included controls for the share of adults (18+) in the population, the share of the population without a high school diploma, the share of non-white individuals in the population, unemployment rate, poverty rate, non-mandatory PDMP legislation indicator, and state, quarter, year, and census-division-by-year indicators.
3. Standard errors were clustered at the state level.

**Figure S4: Sensitivity Analysis Using De Chaisemartin and d’Haultfoeuille (2020) Estimator (Stimulants)**


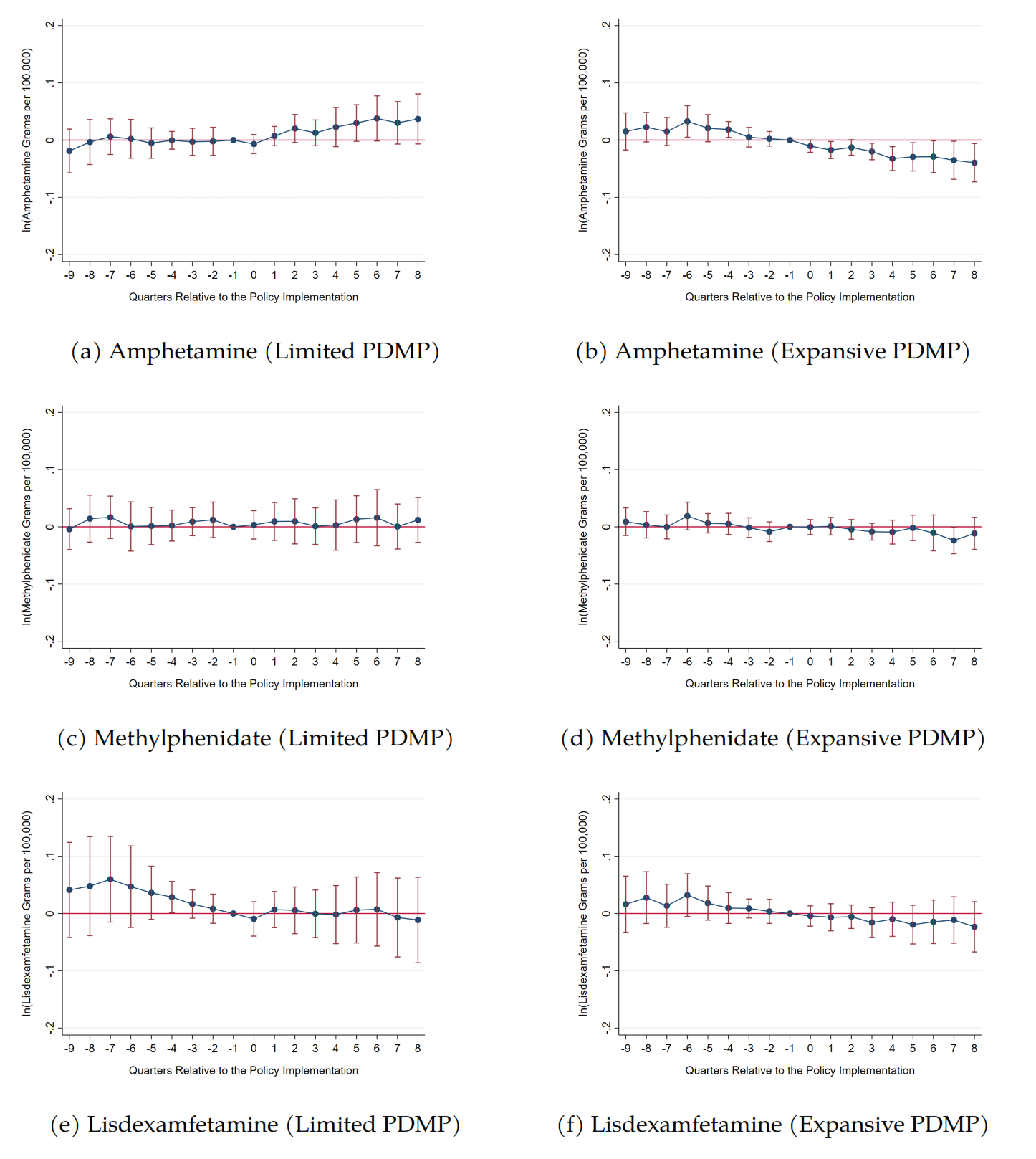


Notes:

1. Limited PDMP use mandate requires prescribers or dispensers to check Prescription Drug Monitoring Program only when prescribing/dispensing opioids or benzodiazepine. Expansive PDMP use mandate is non-specific to opioid/benzodiazepine and requires prescribers or dispensers to check Prescription Drug Monitoring Program when prescribing/dispensing targeted controlled substances in Drug Enforcement Agency Schedule II-V.
2. Estimated coefficient and 95% CIs are reported.
3. De Chaisemartin and d’Haultfoeuille (2020) estimator allowing for different regions (i.e., Census Divisions) to experience differential trends was used. Controls included the share of adults (18+) in the population, the share of the population without a high school diploma, the share of non-white individuals in the population, unemployment rate, poverty rate, non-mandatory PDMP legislation indicator.
4. Standard errors were clustered at the state level.

**Figure S5: Sensitivity Analysis Using De Chaisemartin and d’Haultfoeuille (2020) Estimator (Depressants)**


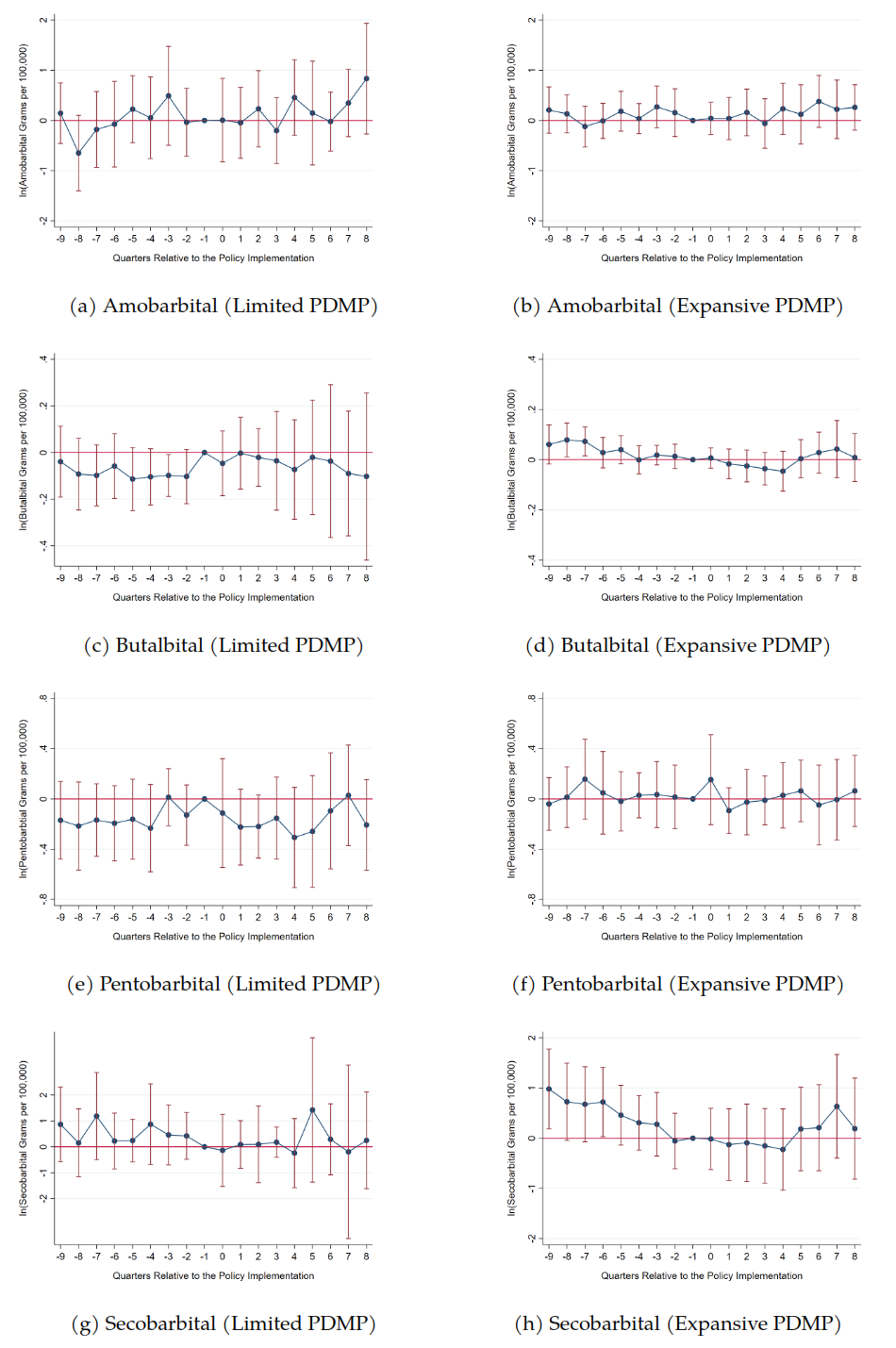


Notes:

1. Limited PDMP use mandate requires prescribers or dispensers to check Prescription Drug Monitoring Program only when prescribing/dispensing opioids or benzodiazepine. Expansive PDMP use mandate is non-specific to opioid/benzodiazepine and requires prescribers or dispensers to check Prescription Drug Monitoring Program when prescribing/dispensing targeted controlled substances in Drug Enforcement Agency Schedule II-V.
2. Estimated coefficient and 95% CIs are reported.
3. De Chaisemartin and d’Haultfoeuille (2020) estimator allowing for different regions (i.e., Census Divisions) to experience differential trends was used. Controls included the share of adults (18+) in the population, the share of the population without a high school diploma, the share of non-white individuals in the population, unemployment rate, poverty rate, non-mandatory PDMP legislation indicator.
4. Standard errors were clustered at the state level.
